# InlL from *Listeria monocytogenes* Is Involved in Biofilm Formation and Adhesion to Mucin

**DOI:** 10.3389/fmicb.2017.00660

**Published:** 2017-04-20

**Authors:** Magdalena Popowska, Agata Krawczyk-Balska, Rafał Ostrowski, Mickaël Desvaux

**Affiliations:** ^1^Department of Applied Microbiology, Faculty of Biology, Institute of Microbiology, University of WarsawWarsaw, Poland; ^2^Université Clermont Auvergne, INRA, UMR454 MEDiSClermont-Ferrand, France

**Keywords:** *Listeria monocytogenes*, internalin, cell-surface protein, biofilm formation, mucins, bacterial adhesion

## Abstract

The bacterial etiological agent of listeriosis, *Listeria monocytogenes*, is an opportunistic intracellular foodborne pathogen. The infection cycle of *L. monocytogenes* is well-characterized and involves several key virulence factors, including internalins A and B. While 35 genes encoding internalins have been identified in *L. monocytogenes*, less than half of them have been characterized as yet. Focusing on *lmo2026*, it was shown this gene encodes a class I internalin, InlL, exhibiting domains potentially involved in adhesion. Following a functional genetic approach, InlL was demonstrated to be involved in initial bacterial adhesion as well as sessile development in *L. monocytogenes*. In addition, InlL enables binding to mucin of type 2, i.e., the main secreted mucin making up the mucus layer, rather than to surface-located mucin of type 1. InlL thus appears as a new molecular determinant contributing to the colonization ability of *L. monocytogenes*.

## Introduction

*Listeria monocytogenes* is the etiological agent of listeriosis, a relatively infrequent but very serious food-borne infections for humans and animals (Schlech, 2000; Vazquez-Boland et al., 2001; Cossart, 2007). This opportunistic intracellular bacterial pathogen is widespread in nature, where it can deal with a wide range of temperature, pH, and osmolarity (Vivant et al., 2013). Actually, this ubiquitous bacterium well-fitted to a saprophytic lifestyle can adapt to different environmental conditions and even switch from commensalism to virulence leading to infections in some special circumstances, namely in immunocompromised people (Gray et al., 2006). Along the food chain, biofilm formation contributes to the survival of *L. monocytogenes* in natural environment and further participates to bacterial persistence and resistance to the cleaning and disinfection procedures in food processing chain lines (Møretrø and Langsrud, 2004; Renier et al., 2011; Giaouris et al., 2014, 2015). Indeed, biofilm bacteria are generally more resistant to environmental stresses, such as organic acids, heavy metals, or antimicrobials resistance, than their planktonic counterparts (Costerton et al., 1995). Surface proteins of *L. monocytogenes* play a key role in facilitating biofilm formation by this pathogen (Renier et al., 2011). As revealed by the most recent proteogenomic analysis (Renier et al., 2012) and without considering integral membrane proteins (IMPs), the *L. monocytogenes* genome actually encodes an impressive total of 147 surface proteins, including 43 LPXTG-proteins and 74 lipoproteins as well as cell-surface appendages. The LPXTG motif allows covalent binding of surface proteins to the cell murein of Gram-positive bacteria and has been found in over 100 bacterial surface proteins (Popowska and Markiewicz, 2004). The number of proteins of this type in *L. monocytogenes* (Buchrieser et al., 2003) is much higher than in many other gram-positive species, e.g., 17 in *Staphylococcus aureus* (Kuroda et al., 2001), 13 in *Streptococcus pyogenes*, 11 in *S. pneumoniae*, 9 in *Lactococcus lactis*, and 3 in *Bacillus subtilis* (Kunst et al., 1997). Besides bacterial adhesion and biofilm formation, those surface proteins interfacing the bacterial cell with its surroundings can be involved in numerous physiological functions, such as cell-wall metabolism, motility, cell-cell communication or transport of numerous substrates, or products as well as virulence (Popowska and Markiewicz, 2004, 2006; Renier et al., 2011; Mariscotti et al., 2014).

Among cell-surface proteins, the genome of *L. monocytogenes* encodes a family of proteins harboring leucine-rich repeats (LRR), called internalins (Bierne et al., 2007). While 35 distinct genes encoding internalins have been identified in the available *L. monocytogenes* genomes, less than half of them have been characterized so far. While InlA and InlB are well-known invasins necessary and sufficient to trigger internalization into epithelial cells (Seveau et al., 2007), InlC was more recently demonstrated to mediate protrusion formation in the course of cell-to-cell spread (Rajabian et al., 2009). A role in pathogenicity has been suggested for the other internalins but, in most cases, their exact molecular contribution remains to be elucidated (Bierne et al., 2007). Most internalins are cell-surface anchored, either upon covalent attachment to peptidoglycan *via* the LPXTG motif (InlA, InlC2, InlD, InlE, InlF, InlG, InlH, InlI, and InlJ, InlK) or cell-wall binding domains such as GW repeats (InlB), but InlC is secreted extracellularly (Bierne et al., 2007). Of note, InlH in *L. monocytogenes* EGD-e results from a recombination event between two genes coding InlC2 and InlD from *L. monocytogenes* EGD. While *L. monocytogenes* EGD corresponds to the original strain isolated by Murray et al. (1926), *L. monocytogenes* EGD-e is basically the very same strain used for sequencing by a European consortium (Glaser et al., 2001) but having divergent subculturing histories (Bécavin et al., 2014). Of these, five internalin genes (*inlA, inlB, inlC, inlJ, InlK*) are involved in the process of invasion or virulence (Gaillard et al., 1996; Raffelsbauer et al., 1998; Bierne and Cossart, 2002; Doumith et al., 2004; Sabet et al., 2008; Neves et al., 2013). Interestingly, 7 internalins contain mucin-binding domain (MucBP), namely InlJ, InlI, Lmo0171, Lmo0327, Lmo0732, Lmo2026, and Lmo2396 (Sabet et al., 2005; Bierne and Cossart, 2007; Bierne et al., 2007; Lindén et al., 2008). The gastrointestinal tract is lined by a protective mucus layer formed by mucin glycoproteins, which acts as a specific barrier to pathogenic microorganisms. Mucin of type 2 (MUC2) is the main secreted mucin making up the mucus layer, whereas mucin of type 1 (MUC1) is cell-surface associated (Lindén et al., 2008). Most human pathogens cause disease by attaching to, and then crossing or disrupting mucosal surfaces. For *L. monocytogenes* InlB, InlC, and InlJ, it has been shown that the LRR was sufficient to bind to MUC2 but not to MUC1 (Lindén et al., 2008).

The *lmo2026* gene was identified in all the *inlGHE*-containing food isolates, suggesting that *lmo2026* might have co-evolved with this locus, which is predominant in *L. monocytogenes* of serovars 1/2a and 1/2c (Chen et al., 2009). Although the function of Lmo2026 in adhesion and virulence processes of *L. monocytogenes* remains to be determined, it was suggested, by screening a bank of signature-tagged transposon mutants in mouse model, that this internalin could affect listerial multiplication in the brain (Autret et al., 2001). However, research is needed to confirm a possible role of Lmo2026 in the crossing of the blood–brain barrier. All-in-all, the function of Lmo2026 remains unknown and does not seem to be related to the internalization process (Bierne and Cossart, 2007). Actually, the term internalin was originally coined upon the functional characterization of Lmo0433 (InlA) and Lmo0434 (InlB) (Gaillard et al., 1991; Dramsi et al., 1995), which are responsible for triggering the internalization of *L. monocytogenes* through specific interaction with eukaryotic ligands such as E-cadherin or c-Met, respectively (Bierne and Cossart, 2002; Seveau et al., 2004). Based on the presence of LRR domains in some potentially secreted proteins, i.e., exhibiting a Sec-dependent N-terminal signal peptide, 33 additional proteins were identified from the available genomes of different *L. monocytogenes* strains (Bierne et al., 2007). While a prototypical internalin domain was tentatively described as comprising a LRR domain flanked by a short α-helical N-terminal cap domain and inter-repeat (IR) domain related to an immunoglobulin fold (Big3), it clearly appeared this succession of domains could not be identified in most cases. In addition, none of the proteins of this family characterized later on (i.e., InlC, InlC2, InlD, InlE, InlF, InlG, InlH, InlI, InlJ, and InlK) has any role in cell internalization *per se*, which somehow indicates that the name of this protein family can be quite misleading and a kind of a misnomer. This is a classical case in which genotype and phenotype are unfortunately confused since homologous genes/proteins do not systematically share the same physiological function. Given the wide host range of *L. monocytogenes* and skill of living in different environments as well as the fact that internalin genes transcription can vary significantly, depending on growth conditions, this prompted us to elucidate the function of Lmo2026 internalin (here renamed InlL), which is not present in non-pathogenic *Listeria* species (Glaser et al., 2001). Following the analysis of the genetic and structural features of InlL, the presence of conserved domains related to adhesins led to investigate its role in adhesion, sessile development and binding to mucins. In this study, we report that InlL is indeed involved in biofilm formation and attachment to MUC2.

## Materials and methods

### Bacterial strains and growth conditions

Bacterial strains used in this study are listed in Table 1. *L. monocytogenes* were grown in Trypticase Soy Broth with 0.6% Yeast Extract (TSBYE) and *E. coli* DH5α in LB (lysogeny broth) (Sambrook and Russell, 2001) at 37°C under shaking. Erythromycin (300 μg/ml for *E. coli* and 1.5 μg/ml for *L. monocytogenes*) or spectinomycin (60 μg/ml) were added to broth or agar media as needed. When necessary, 0.1 mM IPTG (isopropyl-β-D-thiogalactopyranoside) and X-Gal (5-bromo-4-chloro-3-indolyl-β-D-galactopyranoside) (20 μg/ml) were added to agar plates.

**Table 1:** **Plasmids and bacterial strains used in this study**.

**Name**	**Relevant characteristics**	**Source/Reference**
**PLASMIDS**		
pMAD	Thermosensitive allelic replacement vector, *bgaB*, MCS, Amp^R^, Em^R^	Arnaud et al., 2004
pCF430	Derivative of pSW213 carrying containing *araC-P*_BAD_ controlled expression cassette	Newman and Fuqua, 1999
pAT28	Shuttle vector, oritT RK12, oriR pUC, oriR pAMβ1, *lacZ*α, MCS, Spc^*R*^	Trieu-Cuot et al., 1991
pBAL	Derivative of pAT28 with *araC-P*_BAD_ cassette from pCF430, MCS, Spc^R^	This study
pMAD-Δ*inlL*	Allelic replacement vector with Δ*inlL* construct, *bgaB*, Amp^R^, Em^R^	This study
pBAL-*inlL*	Expression vector, with *inlL* CDS (*lmo2026*), Spc^R^	This study
pET-28a	Inducible expression vector, Kan^r^	Novagen
pET-28a-*inlL*	Vector expressing His-tagged InlL	This study
**BACTERIAL STRAINS**		
*E. coli* DH5α	Standard cloning strain	Woodcock et al., 1989
*E. coli* BL21	Strain for protein overexpression	Novagen
*E. coli* BL21 pET-28a-*inlL*	*Strain expressing His-tagged InlL*	This study
*L. monocytogenes* EGD	*L. monocytogenes* wild type *(wt*)	Mackaness, 1964
*L. monocytogenes* Δ*inlL*	Isogenic mutant of *L. monocytogenes* EGD deleted of *inlL* (*lmo2026*)	This study
*L. monocytogenes* Δ*inlL/*pBAL/*inlL*	Complemented strain with *inlL* expressed from the inducible promoter *P*_BAD_	This study

#### Modular architecture and structure modeling of InlJ

To identify conserved motifs, the protein sequence was analyzed using InterProScan v4.3 as the searching tool (Quevillon et al., 2005). The InterPro (IPR) v32.0 database was interrogated (Hunter et al., 2012), which included Pfam (PF) v24.0 (Finn et al., 2014), SMART (SM) v6.1 (Letunic et al., 2009), TIGRfam (TIGR) v10.1 (Haft et al., 2013), PANTHER (PTHR) v9.0 (Mi et al., 2013), SuperFamily (SSF) SCOP v1.75 (de Lima Morais et al., 2011), and PROSITE (PS) v20.7 (Sigrist et al., 2010). LPXTG domain were specifically identified by LPXTG-HMM profile (Boekhorst et al., 2005) and CW-PRED v2.0 (Litou et al., 2008). Signal peptide was predicted using a combinatory approach as previously described (Renier et al., 2012).

For modeling of tertiary structure of the protein domains, analyses were performed from the ORFeus search server available to the academic community *via* Structure Prediction Meta Server (http:/BioInfo.PL/Meta/) (Ginalski et al., 2003), BLAST (Altschul et al., 1990), and FFAS Software (Jaroszewski et al., 2005), as well as using the Swiss Model server (http://swissmodel.expasy.org). For molecular graphics visualization, RasMol v2.7.2.1 was used from RCSB PDB Software (www.rcsb.org/pdb/software-list).

#### DNA isolation and manipulations

Standard protocols were used for recombinant DNA techniques (Sambrook and Russell, 2001). Routine PCR amplifications were performed with DreamTaq (Fermentas), whereas proofreading Pfu DNA polymerase (Fermentas) was used for construction of the deletion mutant and gene complementation. For cloning procedures, DNA fragments, and PCR products were isolated from agarose gels with DNA Gel-Out extraction kit (A&A Biotechnology) according to the manufacturer's instructions. Plasmid DNA was purified from *E. coli* with the Plasmid Miniprep Plus kit (A&A Biotechnology). The procedures for the isolation of plasmid and chromosomal DNA from *L. monocytogenes* were performed as previously described using lysozyme-containing GTE buffer (McLaughlan and Foster, 1998).

#### Construction of an in-frame deletion mutant strain for *lmo2026* in *L. monocytogenes* and gene complementation

Plasmids used in this study are listed in Table 1. *L. monocytogenes* EGD chromosomal DNA was used as the template for the PCR amplification of DNA fragments flanking the CDS (coding DNA sequence) of *lmo2026*. Primers constructed and used in this study are shown in Table 2. Primer pair *lmo2026*-1 and *lmo2026*-2 was used for amplification of a 507 bp fragment immediately upstream of *lmo2026* and primer pair *lmo2026*-3 and *lmo2026*-4 was used for amplification of a 578 bp fragment downstream. A splicing by overlap extension polymerase chain reaction (SOE-PCR) was performed from the two amplicons using primers *lmo2026*-1 and *lmo2026*-4. The resulting PCR product was restriction digested with BamHI and SalI and cloned into the thermosensitive plasmid pMAD using the corresponding restriction sites (Arnaud et al., 2004), yielding pMAD-Δ*lmo2026*. *L*. *monocytogenes* EGD was transformed with this plasmid by electroporation (Monk et al., 2008) and blue-white screening was applied for the selection of gene knock-out (KO) events (Arnaud et al., 2004). The isogenic mutant strain with deleted *lmo2026* gene was called *L*. *monocytogenes* Δ*inlL* and confirmed by DNA sequencing. The non-polar effect of *lmo2026* deletion (mutant PC2) was confirmed by RT-PCR using primer pairs specific for downstream/upstream genes (Figure S1).

**Table 2:** **Primers constructed and used in this study (the complementary sequence are underlined)**.

**Primer**	**Sequence**	**The complementary sequence to**
lmo2026-1	GCGGGATCCCACAGGCAGCCTCCACTTCA	BamHI
lmo2026-2	GTGCTGCAGCGACATTGATTACCAGCAAGAGACATACC	Primer *lmo2026*-3
lmo2026-3	TGTCGCTGCAGCACCAGTTA	
lmo2026-4	GCGGTCGACGCTACTATCGGTTGTTCCTG	SalI
araC-F	GCGGAATTCTGCTACTCCGTCAAGCCGTC	EcoRI
araC-R	GCGGGTACCCAAAAAAACGGGTATGGAGAAAC	KpnI
FP2026	GCGGATCCAGTAGAGTAATTAATAGTCTG	BamHI
F2026	GCGGATCCAACTAAGGACGTGGCACTACA	BamHI
R2026	GCGTCGACATTTTTGCTTGCCCTTGA	SalI
RTC	AGGAACAACCGATAGTAGCG	
RTB	CACATCAGAACTTAGTCCGG	
RTA	TGAATAGCCTCGAGTGTCCA	
L2025	CGCTGGTTGTTCGATGGCAG	
R2025	CATTCTGTACCTGGCGCTGC	
L2026	CCGTGCAATACCTGGATAGT	
R2026	GTAGTGTCTACCGAACCGTC	
L2027	GGACTAAGTTCTGATGTGTCAAGAG	
R2027	GTCACCATTACCATGAGCGG	
hisF2026	CGCGCATATGTCCACTTCATGGATTGACAGG	NdeI
hisR2026	GCGCTCGAGTTAGCTATTTTTTTGCTTTTTAAGTCG	XhoI

For the gene complementation, a new expression vector pBAL was constructed to allow gene expression in *L. monocytogenes* cells at an average and constant level over long periods of time in a manner independent of the medium composition. First, the *araC-P*_BAD_ cassette was PCR amplified using primers araC-F and araC-R from plasmid pCF430 (Newman and Fuqua, 1999). After restriction digestion with EcoRI and KpnI, the fragment was cloned into the high-copy-number *E. coli*–Gram-positive-bacteria shuttle vector pAT28 (Trieu-Cuot et al., 1991). The resulting plasmid construct was confirmed by DNA sequencing and designated pBAL (Figure S2). *lmo2026* was amplified from genomic DNA using forward primers FP2026 or F2026 and reverse primer R2026. The *lmo2026* CDS was PCR amplified using the primer pair F2026/R2026 and cloned into pBAL vector, resulting in the generation of a transcriptional fusion between the *P*_BAD_ promoter and *lmo2026* CDS, i.e., pBAL-*inlL*, as confirmed by DNA sequencing. The pBAL and their derivatives were introduced by electroporation into *L*. *monocytogenes* Δ*inlL* and transformants were selected on BHI plates supplemented with 60 μg/ml of spectinomycin. The obtained strain was designated Δ*inlL*/pBAL/*inlL*. For the wild type (*wt*), mutant and complemented *L. monocytogenes* strains, growth kinetics were determined at 30 and 37°C and restoration of the cell and colony morphotype were checked by microscopic observations. At least three independent experiments were performed for each strain.

### RT-PCR

RNA was isolated using the phenol extraction procedure, resupended in DEPC-treated water and treated with DNAse (Sambrook and Russell, 2001). From there, cDNA was synthesized by PCR using RevertAid™ H Minus First Strand cDNA Synthesis Kit (Fermentas) using sequence-specific primer. In the case of *L*. *monocytogenes* Δ*inlL*, the template used to demonstrate the presence of transcript for the gene upstream—*lmo2025*—was cDNA formed with the use of RTC primer, for *lmo2026*—cDNA formed with the use of RTB primer and for the genes downstream—*lmo2027—*cDNA formed with the use of RTA primer. Primer pair L2025 and R2025 was used for amplification of a 545 bp fragment of *lmo2025* and primer pair L2026 and R2026 was used for amplification of a 546 bp fragment of *lmo2026* and primer pair L2027 and R2027 was used for amplification of a 556 bp fragment of *lmo2027*. Reverse transcriptase-PCR products were electrophoresed in 2% agarose gel (Figure S1).

#### The cell growth, morphology and motility assay

For the wild type, mutant and complemented *L. monocytogenes* strains, growth kinetics were determined at 30 and 37°C and restoration of the cell and colony morphotype were checked by microscopic observations. Motility assay was performed as previously described onto 0.3% BHI soft agar plates incubated at room temperature (Lemon et al., 2007). The diameter of the bacterial colony was measured 24 to 48 h later. At least three independent experiments were performed for each strain.

#### Expression and purification of His-Tagged InlL protein

The InlL protein with a C-terminal hexa-His-Tag was expressed in *E. col*i BL21 using expression vector pET-28a (Novagen). The entire *inlL* CDS was PCR amplified using primers hisF2026 and hisR2026 (Table 2). The amplicon was DNA digested with NdeI/XhoI and cloned into pET-28a prior to electroporation into *E. coli* BL21. The resulting plasmid construct was confirmed by DNA sequencing and designated pET-28a-*inlL*.

For the production of His-tagged InlL (InlL-His6), *E. coli* BL21 carrying pET-28a/*lmo2026* was inoculated in LB supplemented with glucose 0.4% (w/v), 1 mM of AEBSF (4-(2-aminoethyl) benzenesulfonyl fluoride hydrochloride), and 100 μg/mL kanamycin. After overnight growth of the pre-culture at 37°C, a culture was inoculated at 1:100 and further incubated until mid-log phase (OD _600nm_ of 0.6) before adding IPTG (0.5 mM) and transferring the culture at 30°C. At centrifugation (10,000 g, 10 min, 4°C), the bacterial cell pellet was resuspended in sonication buffer (50 mM NaHPO_4_, 500 mM NaCl, 20 mM imidazole, 10 mM β-mercaptoethanol, 0.1% Tween 20, and 10 mM AEBSF). The cells were then disrupted by sonication on ice (VCX-600 ultrasonicator). The cellular debris were pelleted by centrifugation (40,000 g, 1 h, 4°C) and the supernatant was loaded onto a 5 ml Ni-NTA Agarose column (Qiagen). After washing (50 mM NaHPO4, 500 mM NaCl, 40 mM imidazole, and 10% glycerol), the proteins were eluted with imidazole (increments of 50 mM to reach to 1 M). The protein concentration was determined using the Bradford method with BSA (bovine serum albumin) as the standard (Bradford, 1976). Elution fractions containing purified InlL (InlL-His6) were collected and analyzed by SDS-PAGE (12% w/v).

#### Initial bacterial adhesion and biofilm formation assays

Measurement of initial adhesion was based on the crystal violet method as described earlier (Renier et al., 2013). The bacterial culture was adjusted to 1.5 (OD_600nm_) in sterile TSBYE medium and loaded into the wells of a flat-bottom 96-well polystyrene microtiter plate prior to static incubation at 30°C for 1 h.

Quantification of biofilm production in plastic microtiter plates was based on the previously described method (Djordjevic et al., 2002). Briefly, the wells of a sterile flat-bottom 96-well polystyrene microtiter were inoculated from overnight culture adjusted to 0.01 (OD_600nm_) in sterile TSBYE and incubated aerobically at 30°C. At different time points (1, 4, 24, 48, and 72 h), the wells were emptied and washed with sterile distilled water. After fixation with methanol, the wells were emptied and air dried, before adding a crystal violet solution (Gram-color staining set for microscopy; Merck) for 5 min. After washing, wells were air dried and the bound dye solubilized with an aqueous solution of acetic acid The absorbance (Abs) in each well was measured at 570 nm using a microtiter plate reader (Tecan Sunrise 96-well Microplate Readers). At least three independent experiments with at least two repeats each were performed for each listerial strain.

To test the ability of bacterial cell adhesion to the surface of the microtiter plate in the presence of InlL-His6, a competitive assay was performed. To a series of wells, 20 μl of InlL-His6 (30 μg/well) or BSA in phosphate buffered saline pH 7.4 (PBS) (30 μg/well) was added. After incubation for 15 min at 30°C, an overnight bacterial culture was added into each well as described above for the biofilm formation assay and incubated 4 and 24 h.

#### Microscopic observations

The morphology of the cells was observed using a phase-contrast microscope. For electron microscopy observations, the bacterial cells were collected from mid-log phase culture on Millipore HA filter. The cells were fixed for 30 min in 4% paraformaldehyde, washed three times in PBS buffer (pH 7.4) then dehydrated using a series of 15 min incubations in 25, 50, 75, and 100% ethanol and then allowed to air dry for 30 min. The preparations were coated with gold and viewed in LEO 1430VP scanning microscope. The analysis were performed from at least three independent experiments and covered at least 20 electron micrographs.

#### Binding assay of InlL to mucins

Western blotting was performed as previously described (Popowska et al., 2009) in accordance with the manufacturer's recommendations (QIAGEN® Ni-NTA Membrane Protein Kit Handbook). Briefly, purified InlL (InlL-His6) was separated on a 12% SDS-PAGE gel, and electrophoretically transferred to a PVDF membrane (ImmunBlot 0.2 μm polyvinyldifluoride Membrane). The PVDF membrane was blocked with 3% v/v skimmed milk in PBS before incubation in blocking reagent TBS (Tris-buffered saline)/Tween containing anti-His monoclonal primary antibody conjugated with alkaline phosphatase (dilution 1/1000). After washing with TBS/Tween, the PVDF membrane was treated with NBT/BCIP (nitroblue tetrazolium / bromochloroindolylphosphate).

MUC1 from bovine submaxillary glands or MUC2 from porcine stomach were mixed (1 mg/ml) in PBS (pH 7.4) with increased amount of purified InlL (InlL-His6, 0.324 μg/μl): 5, 10, 15, 20, and 25 μl and bovine serum albumin (BSA) (0.4 μg/well) the stock solutions—5 mg/ml in phosphate buffered saline pH 7.4 (PBS), in Eppendorf tubes in total volume of 200 μl. For control of non-specific binding, purified InlL (1.5 μg/ml) was mixed with BSA (1 mg/ml). Samples were incubated for 2 h at 4°C in the presence of the protease inhibitor (AEBSF) at a concentration of 1 mM. The mixtures were then harvested (8,000 × g, 5 min, 4°C) and the supernatant fraction (InlL unbound to mucin) was mixed with SDS-PAGE sample buffer (unbound InlL-His6 to mucin). The pellet fraction (InlL bound to mucin) was first washed twice in PBS to remove unbound material and was resuspended in SDS-PAGE sample buffer. Bound and unbound of purified InlL (InlL-His6) to mucin was visualized by Coomassie staining after SDS-PAGE and additionally by Western blot analyses. Coomassie stained SDS-PAGE were visualized with ImageQuant™ 300 and Western blots with ImageQuant™ TL software for densitometric analyses, respectively. Molecular weight markers were run in parallel (Page Ruler™ Prestained Protein Ladder, Fermentas). At least three independent experiments were performed for each mucin.

#### Statistical analysis

The Student *t*-test was used to compare values. Only *P*-values < 0.05 were considered to be statistically significant.

## Results

### Genetic and structural features of *lmo2026* encoding an internalin of the class I, InlL

The *lmo2026* gene is located in the genome of *L. monocytogenes* adjacent but in the opposite transcription direction to the *nadBCA* operon dedicated to the biosynthesis of nicotinamide adenine dinucleotide (Foster and Moat, 1980; Begley et al., 2001) (Figure S3). On the other side and in the same transcription direction, the *lmo2027* gene encodes an uncharacterized protein belonging to the class III internalins, which are soluble extracellular proteins, including InlC (Bierne et al., 2007). A putative promoter as well as two transcription terminators located upstream and downstream of *lmo2026* could be identified, suggesting a monocistronic genetic organization (Toledo-Arana et al., 2009) (Figure S3).

**Figure 1:**
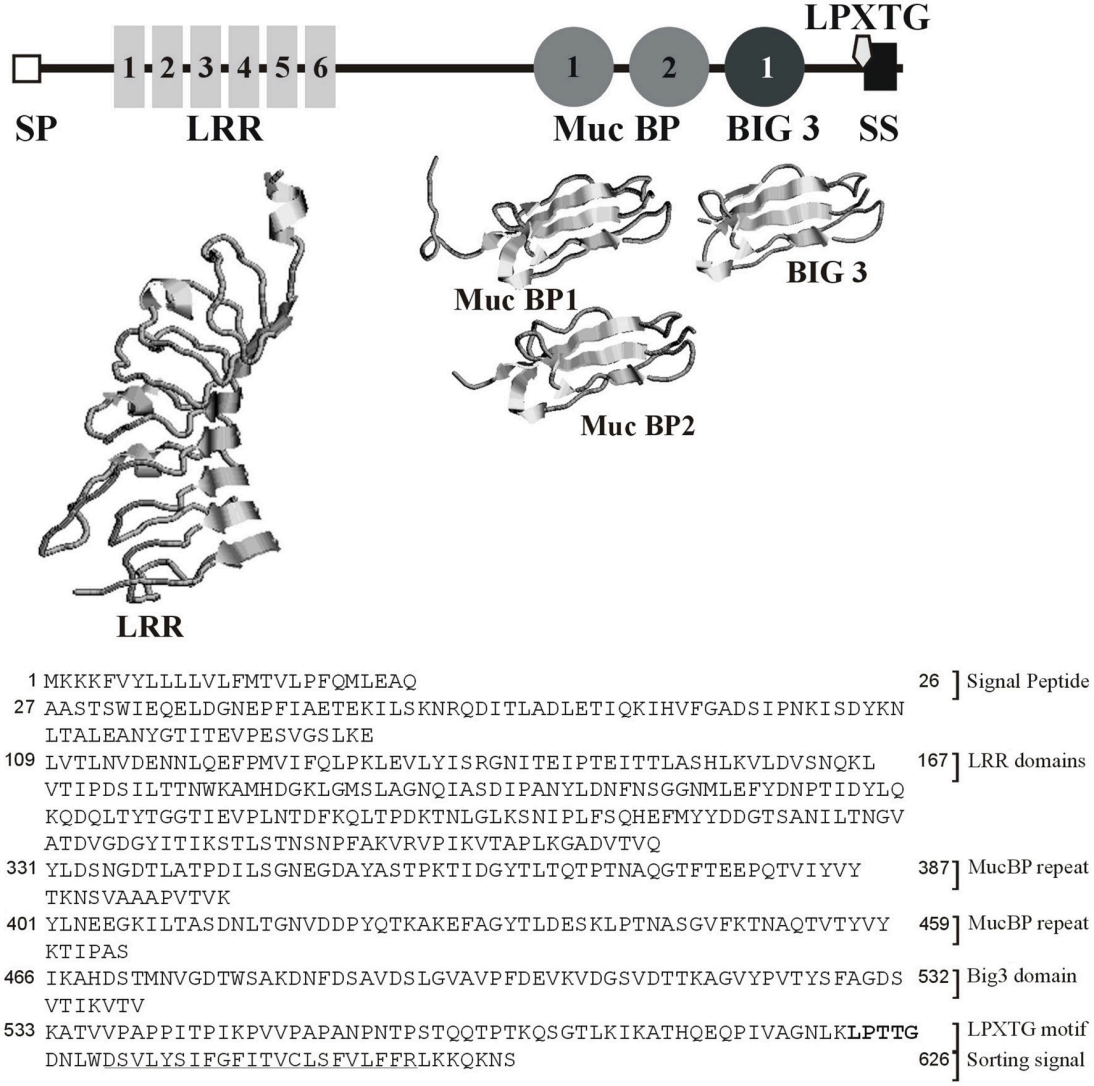
**Modular architecture of InlL based on similarity search for the domain organization and 3-D modeling of the LRR, MucBP, and Big3 conserved domains**. SP, signal peptide; LRR, Leucine-rich repeat domain; MucBP, mucin-binding protein domain, Big3, Bacterial Ig-like domain of group 3; LPXTG, LPXTG domain; SS, sorting signal.

Sequence analysis revealed *lmo2026* encodes a protein of 626 amino acid residues, which exhibits a cleavable N-terminal signal peptide (SP) of 27 amino acid residues. In addition, four distinct types of conserved domains could be identified (Figure 1), namely (i) a LRR domain (PTHR23155: *E*-values = 1.6 × 10^−18^), (ii) two MucBP domains, i.e., MucBP1 (IPR009459, PF06458: *E*-values = 1.2 × 10^−23^) and MucBP2 (*E*-values = 1.1 × 10^−20^), (iii) a bacterial Ig-like domain of group 3 (Big3; IPR011080, PF07523: *E*-values = 3.5 × 10^−24^), and (iv) a C-terminal LPXTG motif responsible for covalent attachment to the cell-wall peptidoglycan (IPR001899, TIGR01167: *E*-values = 2.4 × 10^−2^), further confirmed by LPXTG-HMM and CW-PRED at position 588–626 (Renier et al., 2012). The 3-D modeling of the LRR domain (59–240) revealed it consisted of six repeats and demonstrated significant similarity respective to the fold of protein belonging to the LRR family (Figure 1). Similar observation could be made for MucBP1 (322–402) and MucBP2 (392–464) as well as Big3 (466–531). Considering both the presence of a SP and a LPXTG domain, Lmo2026 is synthesized in the form of a pre-pro-protein, which after complete maturation would result in a mature protein of 60.6 kDa with a *p*I of 4.65. Altogether, Lmo2026 thus clearly belongs to the class I LPXTG-internalins as InlA (Bierne et al., 2007) and has been named InlL (internalin L).

### The role of InlL in cell growth, morphology, and motility of *L. monocytogenes*

No significant difference in the growth rates of *wt*, Δ*inlL* and complemented strains Δ*inlL*/pBAL/*inlL* could be observed, which were at similar levels at the 30 and 37°C, namely with the generation times of ~80 and ~45 min, respectively. No phenotypic differences between the studied strains were detected with respect to hemolytic activity on blood agar plates, growth in “MICROBAT—Listeria Identification System 12L” (Oxoid) or on Oxoid Chromogenic Listeria Agar. With diameters of ~10.5 mm for the bacterial colonies of the *wt* and mutant strains on soft agar, the motility assay could not reveal any significant differences either. Bacterial cells were also examined by electron microscopy when the cultures were in the logarithmic and stationary phases of growth (OD_600nm_ of 0.8 and 1.5, respectively). These microscopic observations revealed similar morphology and size in the different growth phases, bacterial cells appearing longer in the stationary phases (increase by ~10%) than in the exponential growth phases for all studied strains (Figure S4).

### InlL is involved in initial adhesion and biofilm formation in *L. monocytogenes*

**Figure 2:**
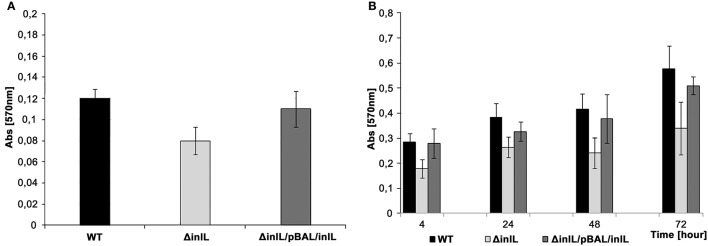
**Initial bacterial adhesion and biofilm formation of *L. monocytogenes wt*, the isogenic *inlL* mutant and the complemented strain at 30°C**. **(A)** Initial adhesion assay based on crystal violet staining. **(B)**. Biofilm formation at different stages of sessile development assayed with the crystal violet method. *L. monocytogenes* EGD *wt* (black bar), Δ*inlL* (light gray bar), and Δ*inlL*/pBAL/*inlL* (gray bar).

Considering the conserved domains of InlL and its potential role as an adhesin, the involvement of InlL in initial bacterial adhesion and sessile development was investigated in the studied *L. monocytogenes* strains. The initial bacterial adhesion was significantly reduced for *L. monocytogenes* Δ*inlL* in contrast to wild type and strain with complementation (Figure 2A).

In investigating biofilm formation at 30°C, significant differences between the *L*. *monocytogenes* Δ*inlL* and *wt* strains were observed regarding the adhered sessile biomass in the course of sessile development (Figure 2B). Indeed, the amount of sessile biomass for the *inlL* mutant was significantly reduced up to 72 h of sessile growth compared to *L*. *monocytogenes wt*. Importantly, no significant difference between the maximum specific growth rates, cell of morphology or motility of *L. monocytogenes wt* and *inlL* mutant strains could be found. Upon complementation with pBAL-*inlL*, biofilm formation was fully restored (Figure 2) and demonstrated that InlL was involved in sessile development of *L*. *monocytogenes* and played a role in attachment to an abiotic surface.

**Figure 3:**
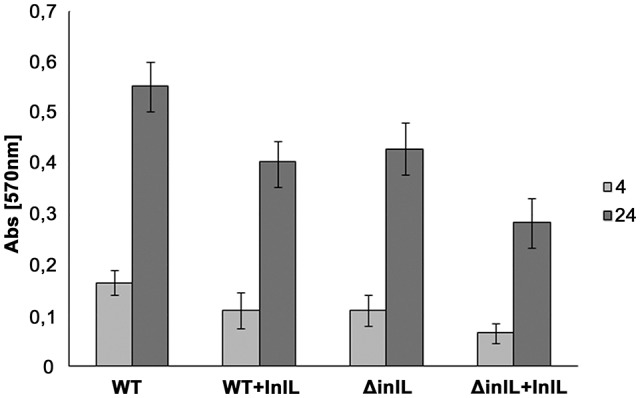
**Competitive adhesion assay of *L. monocytogenes* strains in the presence of purified InlL during biofilm formation process**. Bacterial adhesion to the microtiter plate surface was evaluated in the absence and presence of purified InlL (i.e., +InlL) after 4 h (light gray bar) or 24 h (gray bar) of aerobic growth at 30°C. EGD: *L. monocytogenes* EGD *wt*; Δ*inlL*: isogenic mutant of *L. monocytogenes* EGD (*lmo2026* deletion). At least three independent experiments with at least two repeats each were performed for each listerial strain. See the Material and Methods section for experimental details.

In a competitive assay, purified InlL was added to the wells of the microtiter plate prior to inoculation of *L. monocytogenes* cells (Figure 3). Protein InlL with a C-terminal hexa-His-tag was expressed in *E. col*i BL21, purified on a Ni-NTA Agarose column (Qiagen) and eluted with 100 mM imidazole. The concentration of the purified protein was 1.5 mg/mL. Analysis of fractions by SDS-PAGE using 12% (w/v) polyacrylamide separating gels demonstrated a major protein of approximately 70 kDa, which was shown to be InlL-His6 by Western Blot detection (Figure S5). For further experiments, purified InlL from fractions 4 and 5 were used (Figure S5). The addition of InlL caused a steady decline of adhered biomass of both the *wt* and deletion mutant strains compared to culture in the absence of InlL. Relative to the adhered biomass in the absence of InlL, the percentages of sessile biomass were reduced by around 12 and 19% for *L. monocytogenes wt* and *inlL* mutant respectively after 4 h of biofilm formation in the presence of purified InlL (Figure 3); After 24 h of sessile development, the percentages of sessile biomass were still significantly below those observed in the absence of purified InlL, i.e., around 27% for *L. monocytogenes wt* and 34% for *L. monocytogenes* Δ*inlL*. This significant difference in the formation of biofilm after 24 h indicated InlL bound to the surface of the microtiter plate wells thereby partly blocking bacterial adhesion and possibly the access of other *L. monocytogenes* surface proteins participating in biofilm formation.

### InlL exhibits binding ability toward MUC2

**Figure 4:**
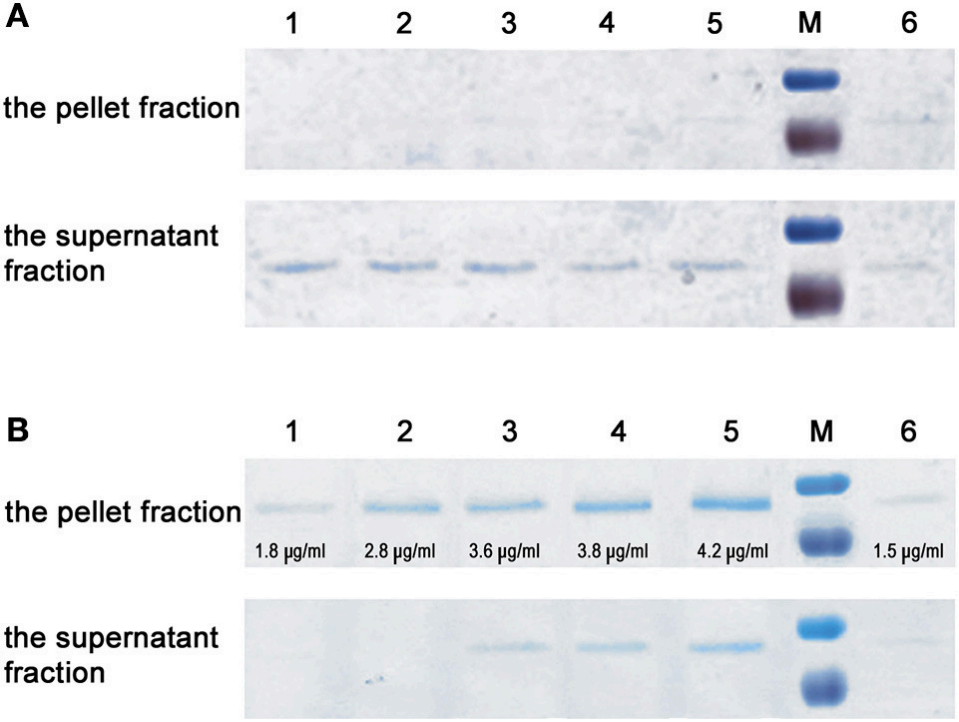
**Interaction of InlL with mucin**. **(A)** Interaction of purified InlL (InL-His6) with MUC1. **(B)** Interaction of purified InlL with MUC2. The pellet fraction (InlL bound to mucin). The supernatant fraction (InlL unbound to mucin). Lane 1-5, the reaction samples of MUC1or MUC2 with added, in increasing amounts, of purified InlL. The concentration of protein bound with MUC2 (B, the pellet fraction) in individual wells, as calculated by densitometry, are as follows: 1–1.8; 2–2.8; 3–3.6; 4–3.8; 5–4.2 μg/ml. Line 6, purified InlL (1.5 μg/ml). M—molecular weight markers standard (Page Ruler™ Prestained Protein Ladder, Fermentas: 100; 70 kDa).

Considering the presence of MucBP1 and MucBP2 domains, the possibility that InlL binds mucin was further tested. The binding ability of InlL to membrane-bound MUC1 and extracellularly secreted MUC2 was investigated. Respective to MUC1, InlL could not bind (Figure 4A). In fact, InlL could only be detected in the supernatant fraction whatever the concentration of protein tested. However, with increasing amounts of InlL, the amount of InlL associated to MUC2 increased as confirmed by densitometric measurements (Figure 4B). When analyzing the supernatant, InlL could only be detected in samples where the saturation point was reached (Figure 4B). The maximum amount (saturation point) of protein InlL associated with 1 mg MUC2 was achieved at protein concentration of ≈4 μg/ml. Those results clearly demonstrated the ability of InlL to bind MUC2, i.e., the mucin released from the surface of eukaryotic cells, rather than to surface-located MUC1.

## Discussion

*L. monocytogenes* encodes an impressive number of proteins belonging to the internalin family (Bierne et al., 2007). This investigation allowed characterizing another member of this family, InlL, which plays a role in the colonization ability of *L. monocytogenes*. Among characterized listerial proteins exhibiting LRR domains, InlJ is the only one previously shown to play the role of an adhesin (Sabet et al., 2008). Bacterial adhesins are specialized cell surface proteins involved in adhesion to abiotic surfaces and/or recognizing specific components at the surface of a host cell or biological tissues, such as cell-surface receptors or extracellular matrix proteins (Chagnot et al., 2013). Together with InlJ and InlL, seven internalins contain MucBP in *L. monocytogenes*, namely InlI (Lmo0333), Lmo0171, Lmo0327, Lmo0732, and Lmo2396. As reported here for InlL, specific binding to MUC2, the major component of intestinal mucus, but not to MUC1, the membrane-bound mucin, was further demonstrated for InlJ but also for InlB and InlC (Lindén et al., 2008). Surprisingly enough, the MucBP domain only present in InlJ did not seem to be required for mucin binding, whereas the LRR domains were suggested to be sufficient for binding to the MUC2. Nonetheless, the involvement of MucBP and LRR domains in the direct interaction with mucin has never been demonstrated in internalins and would require further in-depth investigations of the structure-function relationships.

Interestingly, InlL is absent from non-pathogenic *Listeria* species but also from most sequenced strains of *L. monocytogenes* (Doumith et al., 2004; Bierne et al., 2007). Searching for new genes potentially involved in the pathogenicity of *L. monocytogenes* following a STM (signature-tagged mutagenesis) approach (Autret et al., 2001), an attenuated strain of *L. monocytogenes* was recovered where transposon insertion occurred immediately upstream of *lmo2026* CDS but no functional characterization had been carried. Transcriptomic analysis of *L. monocytogenes* genes expression profile in a mouse model revealed *lmo2026* was up-regulated 24–48 h post-infection (Camejo et al., 2009), whereas a knockout mutant was affected only slightly in its invasion capability (Schauer et al., 2010). While interactions with mucins are require for many enteric pathogens to cause infection (Lindén et al., 2008), the contribution of these internalins to the physiopathology of *L. monocytogenes* still requires in-depth investigations in line with the secretion and dynamics of the different mucins along the gastro-intestinal tract.

*L. monocytogenes* is a zoonotic foodborne opportunistic pathogen primarily circulating within the biosphere, e.g. in the soil, farm, ruminants, or food-processing environments (Vivant et al., 2013). In other words, understanding the ecophysiology of *L. monocytogenes* necessitates to consider both its lifestyle inside and outside the human host. Considering *lmo2026* was mainly identified in food isolates (Doumith et al., 2004; Chen et al., 2009), it prompted us to investigate its involvement in the process of biofilm formation. InlL was here demonstrated to be also involved in initial bacterial adhesion and sessile development to abiotic surfaces. Except for InlA (Franciosa et al., 2009; Gilmartin et al., 2015), this point has never been addressed for any other internalins. While differential expression could be observed between planktonic and sessile development (Gilmartin et al., 2015), the direct involvement of InlA in biofilm formation remains uncertain (Franciosa et al., 2009). While the notion of virulence factors is often promoted first in *L. monocytogenes*, the involvement of these proteins in other processes outside the host should not be overlooked but reconsidered in line with the ecophysiology of such bacterial species.

In conclusion, InlL has been here identified as a novel adhesin required for the attachment of *L. monocytogenes* to abiotic surfaces, where it further participates in biofilm formation, but also binds to the main secreted mucin making up the mucus layer. Much remains to be learned about the respective contribution of the different uncharacterized internalins to the physiology of *L. monocytogenes* in the course of the infection or saprophytic lifestyle (Gray et al., 2006; Vivant et al., 2013). More specifically, the modular architecture and involvement of the MucBP, IR, cap, or Big3 domains as well as LRR, B, or PKD repeats in the biochemical properties of the different internalins still requires in-depth structure-function analyses (Bierne et al., 2007). While the plethora of surface proteins in *L. monocytogenes* suggests some close complementarities in adaptation to different environmental conditions, understanding the global regulation and control of protein expression requires not only considering the transcriptome (Toledo-Arana et al., 2009) but the various post-transcriptional and post-translational levels, in line with the secretome concept (de Lima Morais et al., 2011; Chagnot et al., 2013).

## Author contributions

MP: Contributed to the establishment and coordination of the collaborations, manuscript design, data collection, data analysis, and drafting and writing of the manuscript. AK and RO: Contributed equally to the data collection. MD: Contributed to writing and editing the manuscript, coordination of research and coordination of the collaborations.

### Conflict of interest statement

The authors declare that the research was conducted in the absence of any commercial or financial relationships that could be construed as a potential conflict of interest.
