# Supplementary material for: InlL from Listeria monocytogenes Is Involved in Biofilm Formation and Adhesion to Mucin
Source: Front Microbiol. 2017 Apr 20;8:660. doi: 10.3389/fmicb.2017.00660 (PMC5397405; doi:10.3389/fmicb.2017.00660)
Supplement: Supplementary file 1 [file Presentation1.PDF]

InlL from *Listeria monocytogenes* is involved in biofilm formation and adhesion to mucin

Magdalena POPOWSKA [1,*], Agata KRAWCZYK-BALSKA [1], Rafał OSTROWSKI [1], Mickaël DESVAUX [2]

[1] Department of Applied Microbiology, Institute of Microbiology, Faculty of Biology, University of Warsaw, Miecznikowa 1, 02-096 Warsaw, Poland.
[2] Université Clermont Auvergne, INRA, UMR454 MEDiS, 63000 Clermont-Ferrand, France.

Supplementary material

Figures with legends

Fig. S1

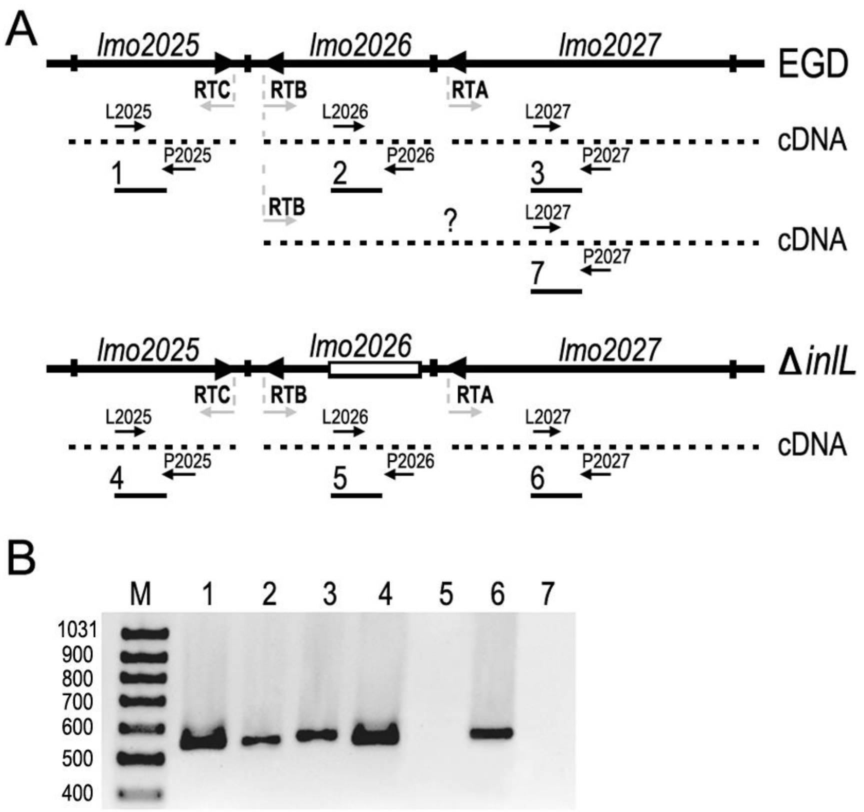

Figure S1. Analysis of transcriptional organisation of the genomic region comprising the *lmo2025*, *lmo2026* and *lmo2027* genes. (A) Scheme for transcriptional analysis of the *lmo2026* genetic locus. The template RNA was isolated from *L. monocytogenes* EGD wt or the *lmo2026* deletion mutant (D*inlL*). Gray arrows indicate the positions of the primers used in RT reactions, broken lines indicate cDNA, black arrows indicate the positions of primers used for PCR and white rectangles indicate the localization of deleted region of *lmo2026*. Black lines labeled 1 through 7 show the positions of the expected or potential products. The RT-PCR product labels correspond to the numbering of the agarose gel lanes in panel b. (B) The products obtained in RT-PCR reactions. The expected size of the amplified fragments of *lmo2025*, *lmo2026* and *lmo2027* was 545 bp, 546 bp and 556 bp, respectively. M: 100-bp ladder molecular weight marker. Control PCRs were performed to confirm the complete removal of DNA from the RNA preparations prior to reverse transcription (data not shown).

Fig. S2

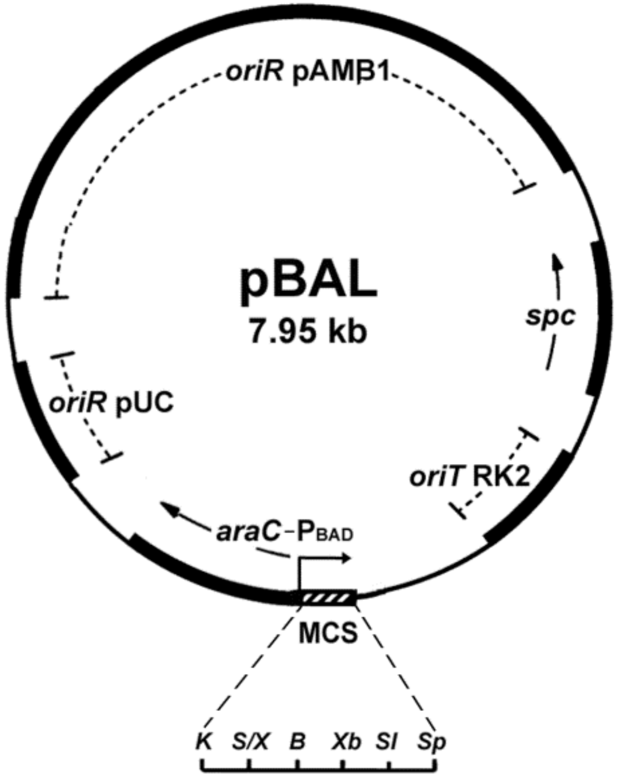

Figure S2. Plasmid map of vector pBAL. *oriR,* origin of replication; *oriT*, origin of transfer; *spc,* spectinomycin resistance gene; *araC*-P_{BAD}, cassette conferring regulator *araC* and promoter of *araBAD* genes of *E. coli*. Arrows indicate the direction of transcription for the multi-cloning site (MCS): B, BamHI; K, KpnI; Sl, Sal; S, SmaI; Sp, SphI; Xb, XbaI; X, XmaI.

Fig. S3

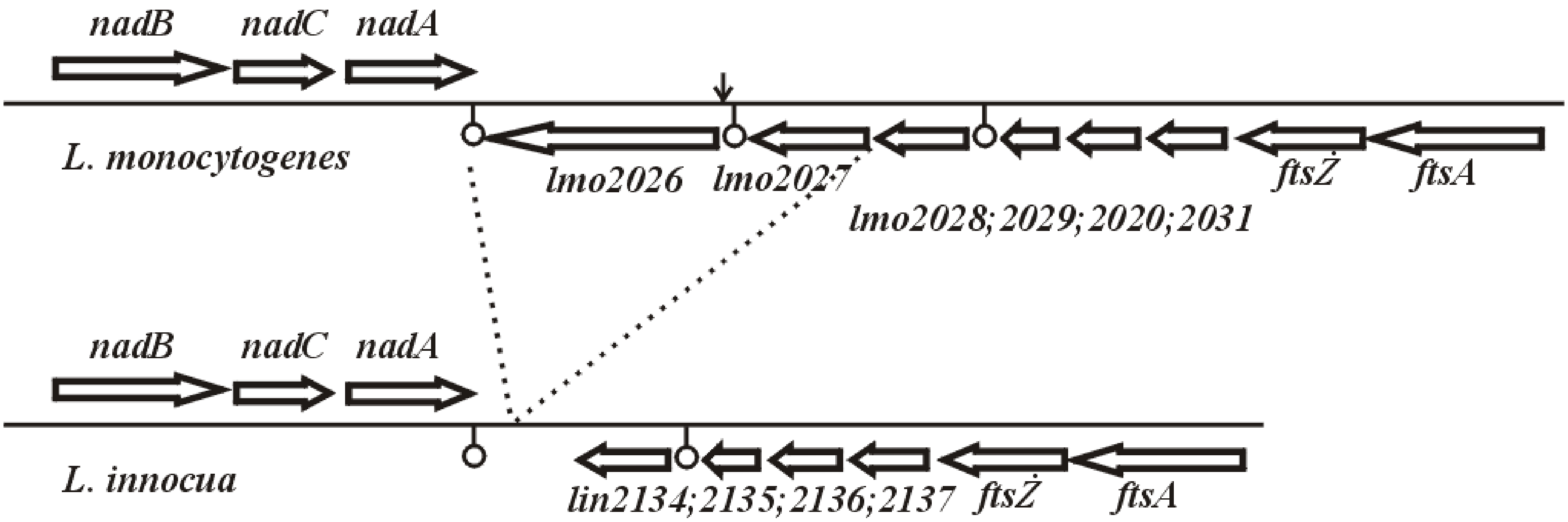

Figure S3. Comparison of the genetic organization of *lmo2026* region in *L. monocytogenes* EGD and *L. innocua*. CDS (coding DNA sequence) and direction of transcription are indicated as well as terminator (⫰) and promoter (↓) of *lmo2026* gene.

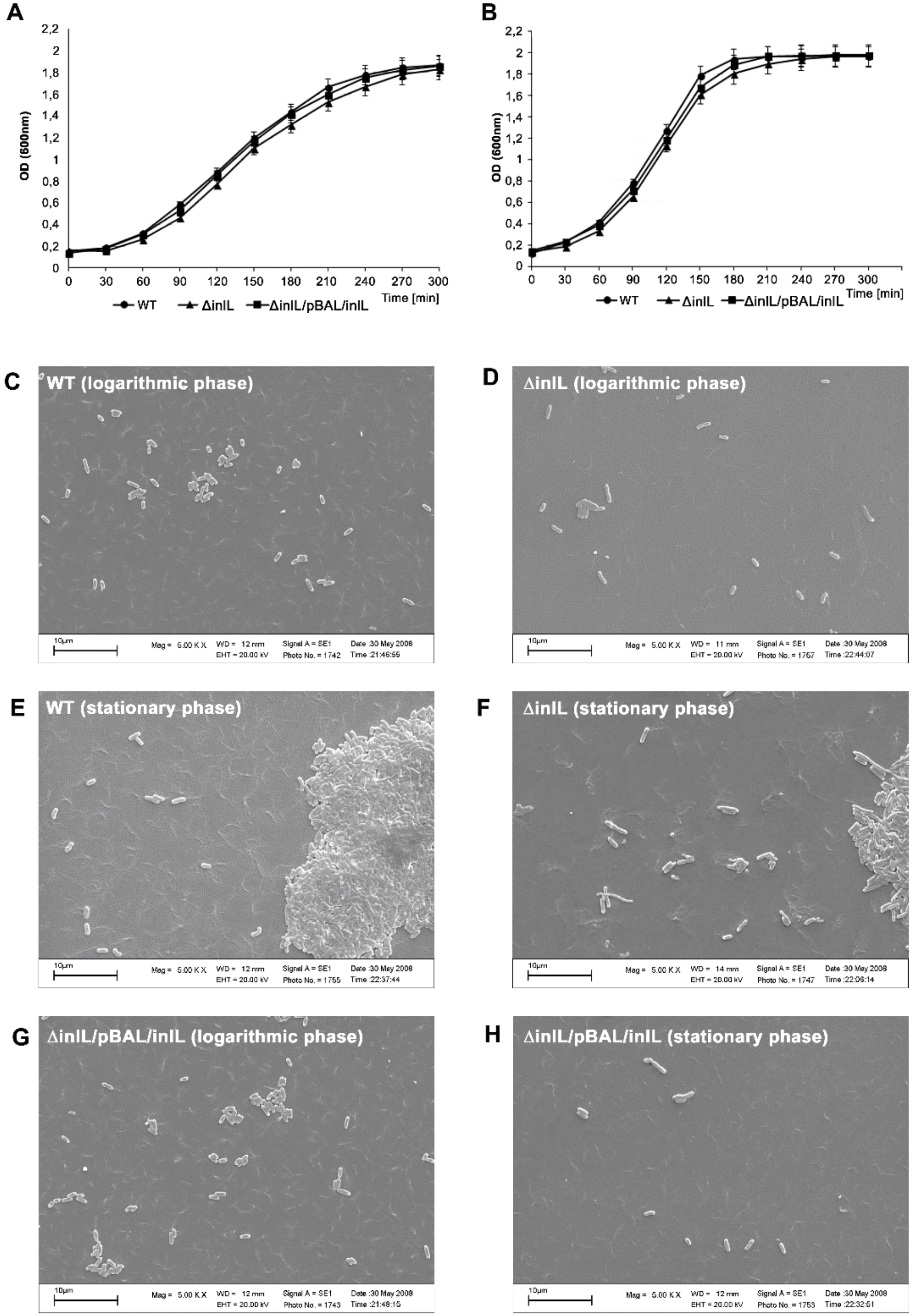

Figure S4. Growth kinetics and microscopic images of *L. monocytogenes* strains. (A): Growth kinetics at 30°C; (B): Growth kinetics at 37°C. Scanning electron micrographs (sample micrographs) (C): *L. monocytogenes* EGD *wt* (logarithmic phase); (D): Δ*inlL* (logarithmic phase); (E): *L. monocytogenes* EGD *wt* (stationary phase); (F): Δ*inlL* (stationary phase). (G): Δ*inlL*/pBAL/*inlL* (logarithmic phase); (H): Δ*inlL*/pBAL/*inlL* (stationary phase).

Fig. S5

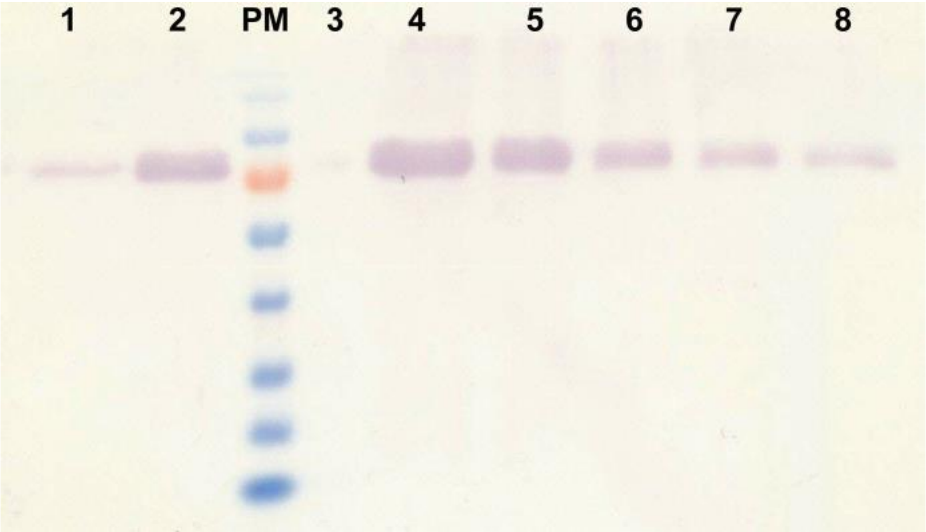

Figure S5. Western blot analysis of purified InlL-His6. Lane PM - molecular weight markers (Page Ruler™ Prestained Protein Ladder, Fermentas: 170; 130; 100; 70; 55; 40; 35; 25; 15 kDa); lanes 1,2, 4-8 - fractions after purification on a Ni-NTA Agarose column; lane 3 - bacterial cell sonicate of a culture of *E. coli* BL21 containing the empty pET-28a vector. For further experiments, fractions 4 and 5 were used.
